# A Rare Thrombophilic Occurrence: Dural Venous Sinus Thrombosis in a Patient with Significant Family History of Protein S Deficiency

**DOI:** 10.7759/cureus.13866

**Published:** 2021-03-13

**Authors:** Eluwana A Amaratunga, James Kamau, Emily Ernst, Richard Snyder

**Affiliations:** 1 Internal Medicine, St. Luke’s University Health Network, Easton, USA; 2 Internal Medicine, St. Luke's University Health Network, Easton, USA

**Keywords:** protein s deficiency, dural venous sinus thrombosis, thrombophilia, cerebral venous sinus thrombosis (cvst), hereditary protein s deficiency, headache

## Abstract

Protein S is a potent anticoagulant that downregulates thrombin formation and is a vitamin K-dependent glycoprotein which is primarily synthesized in the liver. A deficiency in this protein or decreased activity, as seen in hereditary protein S deficiency, can lead to life-threatening thrombosis. Hereditary protein S deficiency is a rare disease as listed by the National Organization for Rare Disorders (NORD). It is known to cause venous as well as arterial thromboembolic events commonly occurring in the deep leg and pelvic veins. Dural venous sinus thrombosis is a rare consequence of protein S deficiency and is associated with a risk of increased morbidity and mortality. We report a case of dural venous sinus thrombosis in a patient with a family history of protein S deficiency in nine family members.

A 53-year-old female presented to the ED with a three-day history of persistent left-sided headache, left facial numbness with tingling, and photophobia. She denied any visual disturbances, slurring of speech, and/or unilateral weakness. Some 10 years prior to this episode, she was placed on warfarin therapy for deep vein thrombosis (DVT) of lower extremity, but she discontinued it after three years of treatment without consulting her treating physician. She was taking oral contraceptive pills (OCPs) for two years and discontinued one month ago. She has nine family members with protein S deficiency, but the patient was never screened for a hypercoagulable state.

On admission, her vital signs were within normal limits. Pupils were round and reactive to light, neck was supple, there was a sensory deficit for pinprick on the left V2-V3 distribution, and remainder of the cranial nerves and neurologic examination was unremarkable. CT scan of the head demonstrated a hyper-density within the left transverse and sigmoid sinus suspicious for dural venous sinus thrombosis. This was confirmed by CT angiogram showing a filling defect throughout the transverse sinus and sigmoid sinus extending below the jugular bulb into the superior aspect of the jugular vein.

Intravenous heparin and warfarin were initiated. As the patient had severe trypanophobia and IV heparin required frequent activated partial thromboplastin time (APTT) monitoring, this was later changed to subcutaneous low-molecular-weight heparin and warfarin. Subsequent thrombosis panel showed a reduced protein S activity of 15% and low levels of total and free protein S antigens. She was discharged home with life-long warfarin therapy.

In conclusion, cerebral dural venous sinus thrombosis is a rare and potentially life-threatening condition that can be seen in hereditary protein S deficiency. A high degree of suspicion in young females with worsening headache and neurologic signs and symptoms will help with timely diagnosis and management avoiding serious consequences. In a patient with a family history of thrombophilia, as seen in our patient, screening is important in order to confirm an underlying thrombophilic state. Such testing may have helped our patient regarding education on avoiding potential risk factors for thrombophilia and importance of treatment adherence.

## Introduction

Protein S is a vitamin K dependent anticoagulant that works in conjunction with activated protein C to inhibit activated procoagulant factors V and VIII restricting clot formation [1]. A deficiency in protein S causes a hypercoagulable state with increased risk of thromboembolism. Protein S deficiency is a rare condition as noted by the National Organization for Rare Disorders (NORD) [2]. It can be hereditary or acquired. Acquired protein S deficiencies can occur with oral contraceptive use, estrogen therapy, pregnancy, liver disease, and acute inflammatory processes [3-4].

Dural venous sinus thrombosis, with an overall incidence of 1.32 per 100,000 person years [5], is a known but relatively uncommon occurrence in patients with thrombophilia. Venous congestion from the outflow obstruction is associated with significant morbidity and mortality [6-8], particularly if management is delayed.

We report a case of a patient, with a significant family history of protein S deficiency in nine family members, who developed dural venous sinus thrombosis.

## Case presentation

A 53-year-old female presented with a three-day history of persistent left-sided headache, left facial numbness with tingling, and photophobia. She denied any visual disturbances, slurring of speech, or unilateral weakness. She was initially seen at an urgent care center where a CT of the head was obtained. The patient was directed to the ED for further evaluation and management. Some 10 years back, she was diagnosed with lower extremity DVT, which was found incidentally after she fell on ice. She was treated with warfarin for three years until she self-discontinued the medication. She had been taking oral contraceptive pills (OCPs) for the past two years and discontinued them one month prior to presentation. Additionally, the patient has nine family members with Protein S deficiency; however, the patient was never screened for a hypercoagulable state.

**Figure 1:**
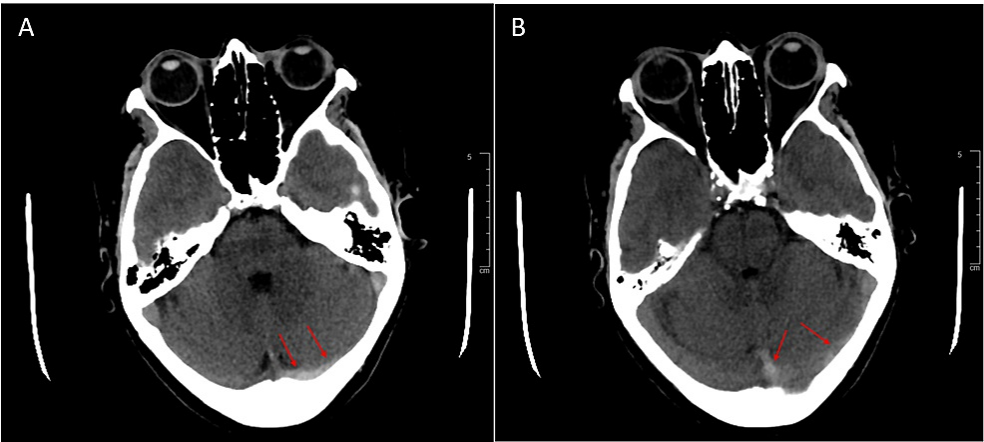
A-B: Noncontrast CT head demonstrating a hyperdensity within the left transverse sinus and left sigmoid sinus.

**Figure 2:**
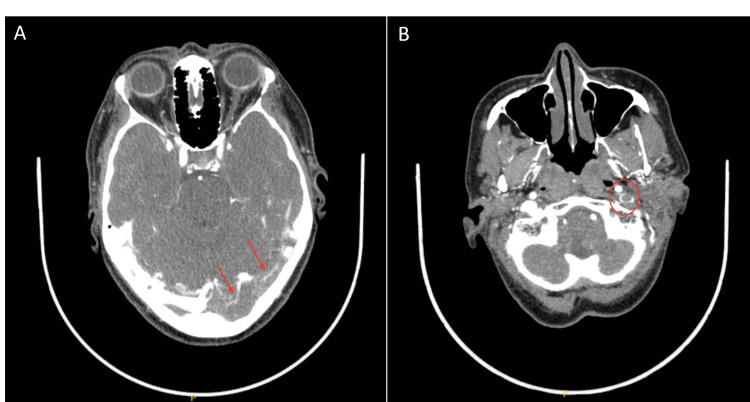
CT angiogram demonstrating a filling defect throughout the left transverse sinus and left sigmoid sinus (A) extending below the jugular bulb into the superior aspect of the jugular vein (B).

Upon arrival to the ED, vital signs were within normal limits. Physical exam was significant for a sensory deficit in the left V2-V3 cranial nerve distribution. The remaining cranial nerve evaluation was normal, and the rest of the neurologic examination was unremarkable. CT of the head demonstrated a hyperdensity within the left transverse sinus and sigmoid sinus suspicious for dural venous sinus thrombosis (Figure 1A,B). This was confirmed by CT angiogram demonstrating a filling defect throughout the left transverse sinus and sigmoid sinus (Figure 2A) extending below the jugular bulb into the superior aspect of the jugular vein (Figure 2B).

The patient was started on IV heparin and warfarin with the intent of transitioning to warfarin once the INR was therapeutic. As the patient had severe trypanophobia and IV heparin required frequent APTT monitoring, this was later changed to subcutaneous low-molecular-weight heparin and warfarin. Subsequent thrombosis panel demonstrated a reduced protein S activity of 15% and low levels of total and free protein S antigen. She was discharged home with life-long warfarin therapy and close follow up with hematology as outpatient. She had complete resolution of her symptoms with anticoagulation.

## Discussion

Protein S, a vitamin K-dependent glycoprotein, is a potent anticoagulant protein causing down-regulation of thrombin formation [1, 9-10]. Plasma protein S levels may show significant interindividual variability depending on age, sex, hormonal status, lipid metabolism, and genetic factors [1, 9]. Approximately 40% of plasma protein S circulates freely, while 60% is bound to complement regulatory factor C4b (C4BP) [9]. Studies have shown that the risk of thrombosis is correlated to a reduction in the free form of this protein [11].

Hereditary or acquired protein S deficiency is caused by either a deficiency of protein S or decreased protein activity, resulting in an increased risk of thrombosis. Hereditary protein S deficiency is autosomal dominant in inheritance. It is caused by multiple mutations involving the PROS1 gene [9, 12-14], with heterozygous mutations resulting in mild disease and homozygous mutations causing severe disease [12]. Hereditary protein S deficiency is divided into three types depending on protein S antigen and activity levels. Type I has low total and free antigen levels with reduced activity, type II has normal total and free antigen levels with reduced activity, and type III has normal total antigen, reduced free antigen, and reduced activity [3, 9, 12].

Hereditary protein S deficiency can present at any age, the majority being among individuals younger than 40-45 years [3]. In rare instances of homozygosity, the initial presentation can occur in infancy as neonatal purpura fulminans [3, 12]. Patients commonly present with thrombosis of various sites. Engesser et al. evaluated 136 members of 12 families with protein S deficiency. Some 74% reported deep vein thrombosis (DVT), 72% superficial thrombophlebitis, and 38% pulmonary embolism either independently or simultaneously [11]. Axillary and mesenteric vein thrombosis [11-12] as well as arterial thrombosis have been rarely reported [3, 10].

As noted in our patient, dural venous sinus thrombosis is a rare manifestation of protein S deficiency, with only a few cases reported in the literature so far [7-8, 15-17]. Although uncommon, dural venous sinus thrombosis is a life-threatening condition with mortality rates being reported between 5% and 30% [8, 18], requiring prompt diagnosis and management. The diagnosis is challenging as headache is the most common presenting symptom [6], which can be confused as other more common etiologies. There needs to be a high degree of suspicion for dural venous sinus thrombosis in any young woman who presents with an unusual, worsening headache with focal neurologic signs [6]. Our patient had a history of migraine headaches and presented with a unilateral headache, with numbness and tingling in the left side of the face, which initially focused our differential diagnoses toward atypical migraine and a stroke/transient ischemic attack. However, with a previous history of DVT of lower extremity and a striking history of venous thromboembolism in nine family members with protein S deficiency, cerebral venous sinus thrombosis became a diagnostic consideration of her headache.

Our patient was managed with IV heparin and warfarin with the intent of transitioning to warfarin monotherapy once the INR became therapeutic (INR of 2-3). Her treatment was further complicated due to severe trypanophobia as she did not tolerate frequent blood drawn for APTT measurements and daily INR measurements. Current evidence-based recommendations on management of dural venous sinus thrombosis include utilizing unfractionated heparin or low-molecular-weight heparin at the time of diagnosis with eventual transition to oral vitamin K antagonists [19]. While novel oral anticoagulants can be used in other thrombotic events associated with protein S deficiency, no studies or data are available to guide the use of these medications in dural venous sinus thrombosis. Therefore, after educating our patient, she was placed on low-molecular-weight heparin with a plan to subsequently transition to oral warfarin therapy prior to discharge, with routine INR measurements to be performed in the outpatient setting. Our patient was successfully treated with no serious end organ damage. The protein S deficiency seen on the subsequent thrombosis panel is likely a reflection of both the patient’s hereditary protein S deficiency as well as the anticoagulant actions of warfarin. The difficulty in interpreting protein S levels while on warfarin therapy has been well described as warfarin is known to cause an acquired protein S deficiency state [3, 12]. Our patient will require life-long anticoagulation with warfarin, further thrombotic panels will have limited clinical utility.

Patients with hereditary protein S deficiency, or other thrombophilia, have a high absolute risk of recurrence of venous thromboembolism, and this risk is further increased after a first spontaneous event [20]. Our patient had a history of DVT, which appeared to be unprovoked. She self-discontinued warfarin after three years of use, likely due to her trypanophobia and reluctance to continue to monitor INR routinely. She was started on oral contraceptives, which is known to affect protein S levels and increase thrombotic risk. A study done by Boerger et al. comparing 21 women taking oral contraceptives to women not taking oral contraceptives, showed that those taking oral contraceptives had lower total protein S and free protein S (functionally active) levels without a significant change in C4BP levels (functionally inactive) [4]. Given our patient’s family history, she would have benefited from screening for thrombophilia as well as patient education on the need for life-long anticoagulation, as well as avoidance of thrombotic risk factors including oral contraception.

## Conclusions

Cerebral dural venous sinus thrombosis is a rare occurrence in patients with protein S deficiency. A high degree of suspicion, especially in young females presenting with unusual headache and neurologic signs and symptoms, will help facilitate timely diagnosis and management of the condition, potentially avoiding serious complications. Furthermore, patients with a family history of thrombophilia presenting with their initial thrombotic event need to be screened for existing thrombophilia. This can help guide the duration of anticoagulant treatment and patient education on potential need for life-long therapy, treatment compliance, and avoidance of risk factors, thus preventing subsequent thrombotic events.
